# Doublecortin regulates the mitochondrial-dependent apoptosis in glioma via Rho-A/Net-1/p38-MAPK signaling

**DOI:** 10.1186/s10020-024-01021-4

**Published:** 2024-12-24

**Authors:** Iqra Nadeem, Zhou Han, Hong Xiaoliang, Seyram Yao Adzraku, Piniel Alphayo Kambey, Kouminin Kanwore, Mu Peipei, Adebayo Oluwafemi Adekunle, Joseph Adu-Amankwaah, Abiola Abdulrahman Ayanlaja, Yi Zheng, Gao Dianshuai, Xiaomei Liu, Yuanjian Song

**Affiliations:** 1 Department of Neurobiology and Anatomy, Key Laboratory of Neurobiology, Xuzhou Medical University, 209, Tongshan Road, Xuzhou, 221004 China; 2 Blood Diseases Institute, Xuzhou Medical University, Xuzhou, 221002 China; 3 Institute of Nervous System Diseases, Xuzhou Medical University, Xuzhou, 221002 China; 4 Department of Physiology, Xuzhou Medical University, Xuzhou, Jiangsu 221004 China; 5 Department of Neurology, Johns Hopkins University School of Medicine, 201 N Broadway, Baltimore, MD 21287 USA; 6 Department of Biology, Illinois Institute of Technology, Chicago, IL USA; 7 Jiangsu Key Laboratory of Immunity and Metabolism, Department of Pathogenic Biology and Immunology, Xuzhou Medical University, Xuzhou, Jiangsu China; 8 Xuzhou Engineering Research Center of Medical Genetics and Transformation, Key Laboratory of Genetic Foundation and Clinical Application, Department of Genetics, Xuzhou Medical University, Xuzhou, China

**Keywords:** Doublecortin, CRISPR/Cas 9, Glioma, Apoptosis, Mitochondria

## Abstract

Doublecortin (DCX) is a microtubule-associated protein known to be a key regulator of neuronal migration and differentiation during brain development. However, the role of DCX, particularly in regulating the survival and growth of glioma cells, remains unclear. In this study, we utilized CRISPR/Cas9 technology to knock down DCX in the human glioma cell line (U251). DCX depletion suppressed cell proliferation and enhanced the pro-apoptotic effects of temozolomide (TMZ) and γ-radiation treatment. DCX knockdown led to the translocation of Bax to the mitochondria and mitochondria dysfunction. Furthermore, DCX deficiency-induced apoptosis took place along with the generation of reactive oxygen species (ROS), which is crucial in triggering mitochondrial membrane depolarization, the release of cytochrome c (Cyt-c), and caspase activation. Importantly, the transcriptional inhibition of DCX downregulated Rho-A, Net-1, and activated p38-MAPK cue, critical for cell survival and proliferation. Subsequent treatment with TMZ and γ-radiation further increased p38-MAPK activity through the decreased expression of Rho-A/Net-1, resulting in a significant reduction in glioma cell migration and invasion. Additionally, intracranial xenograft tumors of DCX-modified U251 cells in nude mice demonstrated inhibited tumor growth. Tumor sections treated with TMZ and γ-radiation exhibited a higher number of TUNEL-positive cells compared to the control group, indicating increased apoptosis. Our finding suggests that DCX depletion reduces glioma cell proliferation and promotes mitochondria-dependent apoptosis by enhancing the chemo and radiotherapy response. Targeting DCX represents a potential therapeutic target for glioma treatment.

## Introduction

Glioma represents one of the most heterogeneous and aggressive malignant tumors that occur in the central nervous system and is characterized by rapid and uncontrolled growth. Currently, surgical excision of glioma is a primary treatment; however, the infiltrative properties of gliomas complicate complete resection often resulting in local recurrence. Even with multimodal treatment approaches, including surgery, post-operative chemotherapy, and radiotherapy, glioma incidence and mortality rates are still increasing (Bush et al. 2017). Due to the poor prognosis and therapeutic interventions, the average overall survival of glioma patients is only 12–16 months (Davis 2018).

Current challenges in glioma treatment include the complex infiltrative growth patterns of tumor and therapeutic resistance which hinder effective apoptosis induction in glioma cells, particularly in response to chemotherapy and radiotherapy. Furthermore, the lack of reliable biomarkers complicates early diagnosis and monitoring of treatment responses, highlighting significant limitations contributing to poor patient outcomes (Wu et al. 2021). Hence, investigating the specific proteins implicated in glioma cell survival and apoptosis, particularly those linked to neuronal microtubule dynamics, may reveal novel biomarkers and therapeutic targets.

Doublecortin (DCX), a 43-kDa microtubule-associated protein (MAP), is indispensable for neuronal migration and differentiation during brain development. As a neurogenesis marker, DCX is highly expressed in the neural progenitor cells of the adult brain, which is typically associated with promoting microtubule assembly and stability in healthy neurons (Gleeson et al. 1999; Horesh et al. 1999). Furthermore, DCX also plays a critical role in regulating cell proliferation and cell cycle (Ayanlaja et al. 2017). Studies have reported overexpression of DCX in various cancers of neuroepithelial origin, particularly in low and high-grade brain glioma, and its correlation with poor prognosis (Daou et al. 2005; Liu et al. 2019; Odrzywolski et al. 2021; Tabrizi et al. 2020; Yáñez et al. 2016). DCX has been identified as a marker of sensitivity and specificity in the invasive margins of glioma (Masui et al. 2008; Reyes et al. 2023). Our previous study also demonstrated that overexpression of DCX and its nucleocytoplasmic transport via the RanGTPase signaling pathway enhanced glioma invasiveness and proliferation (Ayanlaja et al. 2020). These findings suggest the potential role of DCX in glioma progression, however the molecular mechanism underlying this process remains elusive.

Apoptosis is essential for regulating cell proliferation by initiating programmed cell death and contributing to glioma invasiveness and therapeutic resistance (Gousias et al. 2022; Steinbach and Weller 2004). Microtubule-associated proteins have been involved in various diseases including neurodegenerative disorders, and cancers, and are associated with critical cellular processes, notably apoptosis (Breuzard et al. 2019; Nakata et al. 2020). As a microtubule-associated protein, it is plausible that the function of DCX extends beyond regulating glioma cell proliferation potentially contributing to the mechanisms of cell death (Ortensi et al. 2013). Thus, elucidating how DCX influences glioma cell proliferation is pivotal for developing effective therapeutic strategies.

In this study, we tested the hypothesis that DCX directly modulates glioma proliferation and apoptosis both in vitro and in vivo. We showed that downregulation of DCX using a CRISPR/Cas9 technique impaired the proliferation of U251 human glioma cells and induced mitochondria-dependent apoptosis. Further, our data demonstrate that several key proteins, Rho-A, Net-1, and p38-MAPK which are essential for cell proliferation and apoptosis are likely involved in DCX-mediated signaling pathways. Therefore, our studies suggest that DCX is a critical regulator of apoptosis in glioma cells, and may serve as a novel therapeutic target for glioma treatment.

## Materials and methods

### Cell culture

The human glioma cell line U251 was obtained from the Cell Bank of the Chinese Academy of Sciences (Shanghai, China). Cells were cultured in Dulbecco’s Modified Eagle Medium (DMEM) high glucose medium (Thermo Fischer Sci, 11965092) supplemented with 10% fetal bovine serum (Gibco™, 26140079) and 1% penicillin-streptomycin (Sigma-Aldrich, P4333). Media changes were performed every 2 to 3 days to maintain optimal culture conditions. Cells were grown in a humidified 5% CO_2_ incubator at 37 °C and used for up to ten passages, with routine assessments of morphology and viability.

### In vitro treatment with TMZ and the irradiation procedure

The cell line was routinely tested to confirm the absence of mycoplasma contamination (Beyotime, C0301S). TMZ (MCE, HY-17364) was dissolved at a stock concentration of 100 mM in dimethyl sulfoxide and stored at − 20 °C. TMZ was diluted in a culture medium immediately before the treatment of cells to a concentration of 50 µM. The U251 cells were seeded at 1 × 10^5^ cells and treated with gamma (γ) radiation after reaching 80% confluence using a GSR C1 137 cesium gamma irradiator (Gamma-Service Medical, Bautzner, Germany) at a dose of 8 Gy with a dose rate of 1.88 Gy/min. The irradiated cells were cultured in humidified 5% CO_2_ incubators at 37 °C until further analysis. Non-irradiated control samples were treated similarly (i.e., culture medium, transport to the accelerator, and incubation conditions). TMZ was added to the cells directly after irradiation. The cells were harvested 24 h after the treatment and used for further experiments.

## Establishment of DCX CRISPR/Cas9 modified cells

Employing CRISPR-Cas9 technology, U251 cells were genetically engineered to stably express DCX knockdown. A DNA fragment corresponding to the DCX gene was obtained via polymerase chain reaction (PCR) amplification using upstream primer:5’-GGCGATCTGGTGGAGTTCGT-3’ and downstream primer: 5’-TTTGTTAGACGAAGCTTGGGCTGCA-3’. The DNA fragment was ligated into the GV468 vector and the recombinant plasmid was digested with GV468-DCX targeting at human DCX. The digested plasmid constructs were then transfected into HEK293T cells. Puromycin was used to screen for cells expressing DCX. The efficiency of lentivirus infection was assessed through microscopic observation of green fluorescent protein (GFP) expression intensity, while the virus titer was determined using a virus gradient dilution method. The DCX mRNA and protein expression levels were assessed using quantitative real-time PCR and Western blotting, respectively.

## Protein extraction and western blotting

Whole-cell protein was extracted using RIPA cell lysis buffer with protease inhibitor and quantified by the BCA protein kit (Beyotime, P001). Cells were cultured without penicillin for the mitochondrial protein extraction, and the mitochondrial and cytosol fraction was prepared using the mitochondria isolation kit (Beyotime, C3606), per the manufacturer’s instructions. Proteins were separated by 7.5–12.5% SDS-PAGE and transferred to polyvinylidene difluoride membranes (0.45 µM PVDF, Millipore, USA). The membranes were blocked with the skimmed milk for 60 min at room temperature and then incubated overnight at 4 °C with the respective primary antibodies: DCX (Abcam, ab207175); Bax(Abcam, ab216494); Bcl-2 (Abcam, ab196495); Cleaved-caspase-3 (Proteintech, 25546-1-AP); Net-1(Abcam, ab113202); Rho-A (CST, #2117); Phospho-38 MAPK (CST, #9211); p38-MAPK (CST, #9212). GAPDH (Proteintech,10494–1-AP), β-tubulin (Proteintech,66240-1-Ig), and TOM-20 (Abcam, ab56783) were used as an internal reference protein. Membranes were incubated with the secondary antibody IRDye ab 800CW (Vicmed, 926-32210) for 2 h at room temperature. Bands were visualized using the LI-COR Odyssey CLx imaging system, and the intensity of protein expression was measured using ImageJ software.

## qRT-PCR

Total RNA was extracted from the cells with the TRIzol reagent (Invitrogen Life Technologies, 15596-026) as described previously (Jozefczuk and Adjaye 2011). Briefly, the RNA was reverse transcribed into cDNA with a qRT Super Kit (QIAGEN, 204443) in 20-µl reactions. RT-PCR was performed with a LightCycler 480 (Roche), and GAPDH was used to normalize the gene expression. The expression level of the target genes was obtained using the 2−∆∆Ct calculation method. GAPDH forward primer: GGAGCGAGATCCCTCCAAAAT, Reverse primer: GGCTGTTGTCATACTTCTCATGG; Caspase-3 Forward primer: TTTTTCAGAGGGGATCGTTG, Reverse primer: CGGCCTCCACTGGTATTTTA; Net-1 gene forward primer: GAGCCAAGCAATAAAAGAGTTCG, Reverse primer: TGGGACTGTTGACCTGCTAGA; BCL-2 forward primer: GAGGATTGTGGCCTTCTTTG, reverse primer: ACAGTTCCACAAAGGCATCC; Bax forward primer: TTTGCTTCAGGGTTTCATCC, reverse primer: CAGTTGAAGTTGCCGTCAGA.

## Proliferation assays

The MTT and EdU assays were used to measure cell proliferation. For MTT, cells were seeded at 2 × 10^^3^ per well in 96-well plates and cultured in DMEM with 10% serum for 24, 48, 72, and 96 h. After incubation, 0.2 mg/ml MTT (Sigma-Aldrich, M5655) was added to each well and incubated for 4 h. Then, 200 µl of dimethyl sulfoxide was added to dissolve the formazan crystals. The absorbance of the cells was measured at 490 nm with a spectrophotometer (BioTek, Synergy H1). Experiments were conducted in triplicate, and IC50 values were calculated using GraphPad Prism 10. EdU was incorporated into proliferating cells and assessed through a catalyzed reaction with an iClick™ EdU Andy Fluor™ 647 Imaging Kit (Thermo Fisher, C10340). EdU-positive cells were determined and quantified under fluorescence microscopy (Olympus, BX53).

### Cell death detection

Cell death was assessed using the PE Annexin V Apoptosis detection kit with 7-AAD, according to the manufacturer’s instructions (Biolegend, 640934). 1 × 10^7^ cells/ml were resuspended in a binding buffer and stained with 5 µl PE Annexin V and 7-AAD in the dark at room temperature for 15 min. Apoptotic cell numbers were analyzed using a flow cytometer (BD Biosciences) and FlowJo software 7.6.1 (FlowJo, LLC).

## Clonogenic assays

1 × 10^3^ cells were seeded into 60 mm dishes containing 10 ml of DMEM supplemented with 10% FBS. Following two weeks of incubation, colonies were washed with PBS thoroughly, fixed with methanol for 5 min, and stained with Giemsa solution (Beyotime, C0133) for 20 min. Visible colonies (larger than 50 μm in diameter) were manually counted as positive for growth.

## Transwell matrigel invasion assay

24-well Transwell inserts (upper chambers) were pre-coated with 50 µl of diluted Matrigel (Corning, 356237). The upper chamber was seeded with 1 × 10^5^ cells in 100 µl serum-free medium, and 600 µl complete medium was put in the lower chamber. Then, cells were cultured at 37 °C for 24 h, and non-invading cells were removed using a cotton swab. Cell fixation was done with methanol for 15 min, and 0.1% crystal violet was used for staining (30 min at RT). Then, the invaded cells were counted under an inverted microscope. (Olympus, IX73)

### Wound healing migration assay

1 × 10^4^ cells were seeded into a 6-well plate and were cultured until they achieved a confluent monolayer. A scratch was made in each well using a 200 µL sterile pipette tip, and detached cells were removed by washing with PBS. Cells were cultured with a complete medium, and images of the wound healing area were taken at 0 and 18 h with a microscope.(Olympus, IX73).

### Immunofluorescence staining

Cells with and without treatment were seeded into 24-well plates in humidified 5% CO_2_ at 37 °C for 24 and allowed to attain 60–70% confluence. The following day, cells underwent PBS washing and were fixed with 4% paraformaldehyde and permeabilization with 0.1% Triton X-100. Blocking was done using 2% BSA–PBS for 60 min at room temperature, and cells were stained with these primary antibodies at 4 °C overnight: DCX (Abcam, ab207175), Net-1 (Abcam, ab113202), and Cleaved-caspase 3 (Proteintech, 25546-1-AP). Cells were rinsed with PBS and incubated in secondary antibodies Alexa Fluor^®^ 594 (1:500, Thermo Fisher, I14402) for 45 min under dark conditions at 37 °C, and the nucleus was counterstained with DAPI. The coverslips were mounted with an anti-fading mounting medium, and the fluorescent signals were observed with a microscope (Olympus, BX53).

### Cellular ATP content detection

ATP concentration was measured with the ATP Assay Kit (Beyotime, S0026) luciferin-luciferase method. Briefly, 1 × 10^5^ cells were cultured in 6-well plates for 24 h, lysed, and centrifuged at 1000 g for 5 minutes. The pellets were lysed in 200 µL lysis buffer and centrifuged at 12,000 x g for 5 min at 4 °C. Following centrifugation, 100 µL of the ATP detection solution was mixed with the supernatant. The standard curve was produced by determining protein concentration with Bradford assay. Luminescence was detected immediately using a microplate reader (BioTek, Synergy H1).

### Reactive oxygen species (ROS) assay

Cells were seeded on black 96-well plates, and the amount of intracellular ROS production was measured using the Fluorometric Intracellular ROS Kit (Sigma Aldrich, MAK144). 200 µL of Master Mix was loaded in an individual well before incubation for 45 min under 5% CO_2_ at 37 °C. Fluorescence signals were then determined using a microplate reader (BioTek, Synergy H1) at an excitation/emission wavelength of 540/570 nm. The results were expressed in percentage fluorescence relative to corresponding controls.

### Mitochondrial membrane potential (MMP) assay

The mitochondrial membrane potential (MMP) was determined using a JC-1 reagent (Beyotime, China, C2006) following the manufacturer’s instructions. The cells were plated at 1 × 10^5^ cells/dish and treated for 24 h, then were washed with PBS trailed by incubation using the JC-1 probe at 37 °C for 40 min. Images of green and red fluorescence were captured using a fluorescence microscope.

### Mitochondria Staining

Cells were cultured on the coverslips overnight, fixed with 4% paraformaldehyde for 30 min, permeabilized with 0.1% of Triton-X100 for 5 min, followed with MitoTracker™ Deep Red (Beyotime Biotech, C1034, China) staining for 30 min at 37 °C. After the immunostaining, cells were counterstained with Hoechst (Abcam, ab228550) and examined under a confocal microscope (Olympus FV10i).

### Transmission Electron Microscopy (TEM)

The glioma cells were digested, collected in a centrifuge tube, and immobilized using 2.5% glutaraldehyde at 4 ℃ overnight. Cells were washed twice the next day with PBS and fixed using 1% osmium tetroxide. After fixation, the cells underwent gradient dehydration. Infiltrated cells were embedded with resin, sectioned, and stained with 2% uranyl acetate for 5 min. The subcellular structure of mitochondria was observed using transmission electron microscopy (TEM). (Tecnai G2 T12; Hillsboro, OR, USA).

### Caspase 3/7 activity analysis

Cells were collected and incubated for 1 h in the dark with 100 µL Caspase-Glo 3/7 reagent (Promega). Luminescence of each sample was detected by a microplate reader at 485/530 nm. Caspase 3/7 activity was calculated to evaluate fold-changes of U251 DCX knockdown samples relative to control. The results were calculated as representative examples based on three independent experiments.

### Tumor xenograft Orthotopic Model

The Institutional Animal Care and Use Committee of Xuzhou Medical University approved all experimental protocols (approval No. 202009A164). Male BALB/c nude mice (6–8 weeks old) were purchased from Beijing Weitong Lihua Experimental Animal Technology Co., Ltd and were housed under specific pathogen-free conditions. For the initial construction of the orthotopic animal model, 5 mice were used per group to validate the successful implantation of CRISPR/Cas9-modified U251 DCX knockdown glioma cells (5 × 10^5^ cells in 3 µl PBS). The cells were implanted stereotactically into the right basal ganglia of the mice brains using these coordinates (anterior-posterior + 1.0 mm, medial-lateral + 2.0 mm, and dorsal-ventral − 3.0 mm from the bregma and dura) with a 10-µl Hamilton syringe at a speed of 1 µl/min (Huse and Holland 2009). After establishing the model, mice were divided into six groups (10 mice/group), each prepared for treatment and survival analysis. Seven days post-tumor implantation, mice were treated for 5 days with TMZ (50 mg/kg intraperitoneally) and gamma radiation (6 Gy at a rate of 2 Gy/min directed at a tumor site). Mice were sacrificed when they reached a humane endpoint, and Kaplan–Meier survival curves were constructed by plotting the proportion of surviving mice versus time post-treatment. Experimental animals were observed every two days until they showed moribund signs, then the mice were anesthetized, followed by intracardiac perfusion with PBS and formalin. Brains were removed, further fixed in 4% paraformaldehyde, and embedded into an OCT compound (Tissue-Tek).

### Hematoxylin-Eosin and immunostaining

Brain tumors from euthanized mice were collected, and 5 μm-thick sections were prepared. According to standard procedure (Feldman and Wolfe 2014), the H&E Staining Kit (ab245880) was used to stain frozen brain tissue sections. Following cold acetone fixation and antigen retrieval, immunostaining was carried out by incubating section with the Ki-67 primary antibody (1:200; Servicebio: GB111141). Sections were washed with PBS, and a secondary antibody, anti-rabbit IgG conjugated to Alexa 594 (1:1,000; Molecular Probes, Paisley, UK), was added. Nuclear counterstaining was performed by incubation in DAPI for 10 min at room temperature. H&E and Ki-67 stained sections were analyzed under a microscope (Olympus BX53).

### TUNEL staining

Apoptosis in brain tissues was evaluated with the One Step TUNEL Apoptosis Assay Kit (C1090, Beyotime Biotechnology, China). Brain slices were incubated in 0.5% Triton for 10–20 min, then washed in PBS. 50 µL TUNEL reagent was added into each slice as described in the instructions and kept in a dark environment at 37 °C for 2 h. Slices were washed with PBS and stained with DAPI (KeyGen Biotech, A215–10). TUNEL-positive cells were visualized and counted using a fluorescence microscope.

### Statistical analysis

Each experiment was repeated ≥ 3 times to ensure reproducibility. Data are presented as means ± standard deviation. GraphPad Prism^®^ 10.1.2 (GraphPad Software, Inc., San Diego, CA, USA) was used for statistical analysis and graph preparation. For comparisons between two or more groups, the non-parametric Student t-test was used, while analysis of variance (ANOVA) was employed for comparison among multiple groups. Statistical significance was defined as *p* < 0.05. Levels of significance were indicated as *P < 0.05, **P < 0.01, ***P < 0.001, ****P < 0.0001, and NS (not significant).

## Results

### DCX knockdown suppresses glioma cell proliferation

**Fig. 1:**
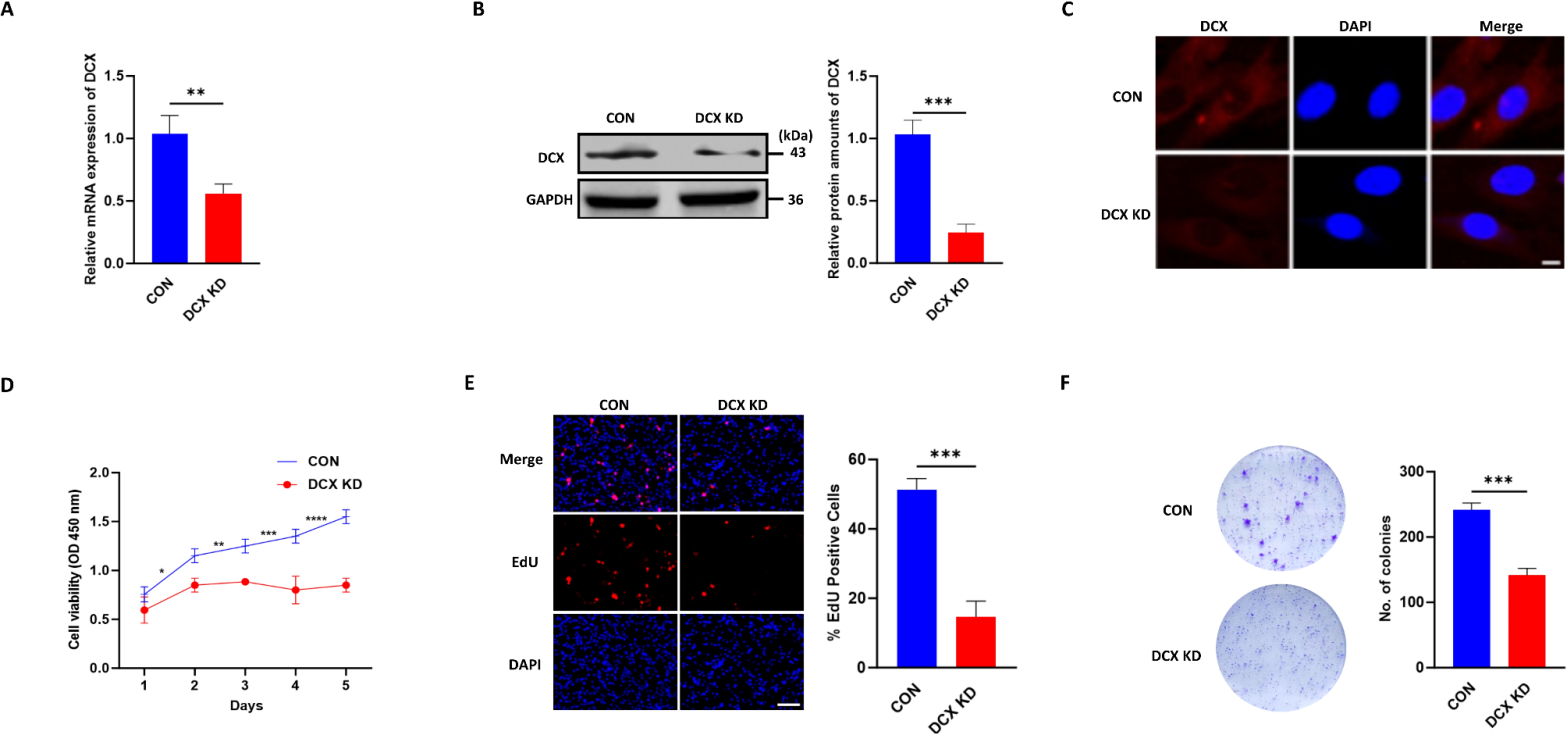
DCX knockdown suppresses glioma cell proliferation. **(A and B)** DCX knockdown by CRISPR/Cas-9 in the U251 glioma cell line was validated using Western blot and qPCR analysis (CON: Blue; DCX KD: Red). **(C)** Immunostaining of DCX in U251 stable cell lines using a DCX-specific antibody (Red) and nuclear counterstain DAPI (Blue). Scale bar, 25 μm. **(D)** MTT assay was used to examine the effect of DCX knockdown on cell viability. **(E)** EdU assays detected the cell proliferation after DCX knockdown. **(F)** Representative photographs of the colony formation assay demonstrated that the smallest number of colonies was observed in the DCX knockdown group compared with control groups: scale bar, 100 μm. All experiments were repeated three times, and data are presented as mean ± SD. ^*^P < 0.05, ^**^P < 0.01^, ***^P < 0.001, ^****^P < 0.0001, compared with control cells

To investigate DCX’s effects on glioma cell survival and growth, we employed the CRISPR/Cas9 technique to stably knock down DCX in U251 cells. The efficacy of DCX knockdown was validated using qPCR, Western blotting, and immunofluorescence staining, which all showed a significant reduction in DCX expression levels in the knockdown group compared with the control group (Fig. 1A-C). MTT assay was used to assess cell proliferation. Silencing of DCX resulted in reduced cell viability at 48, 72, or 96 h (Fig. 1D). Similarly, as demonstrated by the EdU incorporation and colony formation assays, loss of DCX significantly decreases the proliferation and growth of the U251 cells (Fig. 1E, F). These results indicate that DCX knockdown markedly inhibits the proliferation of glioma cells.

### DCX downregulation promotes glioma cell apoptosis

**Fig. 2:**
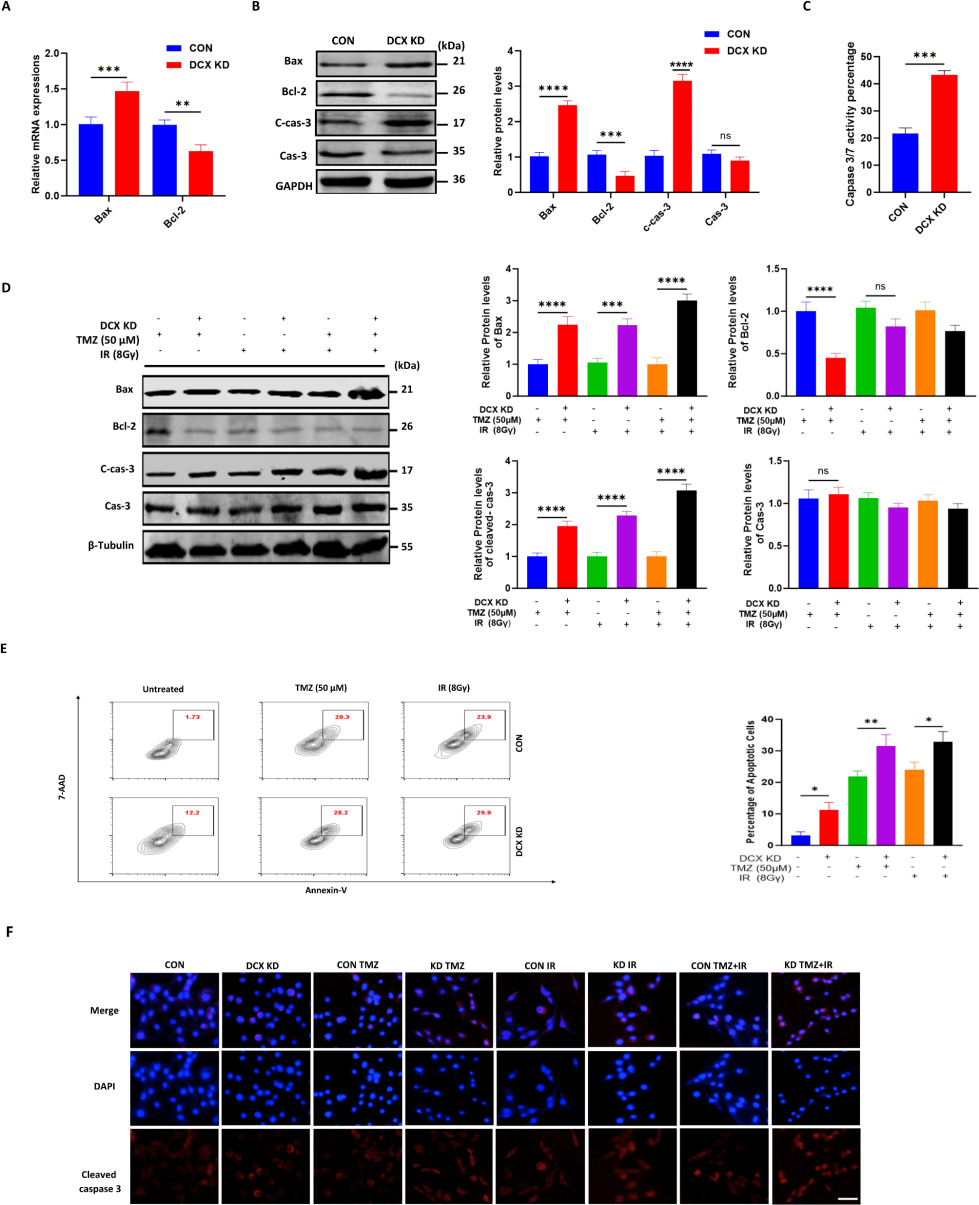
DCX downregulation promotes glioma cell apoptosis. **(A)** The mRNA levels of Bcl-2 and Bax were detected by q-PCR. Values were normalized relative to the β-Actin mRNA levels. **(B)** After DCX knockdown, immunoblot analysis was performed to detect the expression of cleaved caspase-3, caspase-3, Bax, and Bcl-2 (CON: Blue; DCX KD: Red; CON TMZ: Green; KD TMZ: Purple; CON IR: Orange; KD IR: Black). Analyses of protein were performed using Image J. The protein content was normalized against the corresponding GAPDH content. **(C)** The caspase 3 /7 activity was analyzed using the Caspase-Glo 3/7 assay. **(D)** U251 cells were treated with indicated concentrations of TMZ and γ-radiation, and Western blotting was conducted to detect the expression of apoptotic proteins. β-tubulin was used as an endogenous control. **(E)** Cells were analyzed for apoptosis with Annexin-V/7-AAD by flow cytometry. **(F)** Cleaved caspase-3 immunofluorescence staining of U251 cells pre and post-treatment for 24 h, scale bar, 50 μm. For all experiments, data represent the mean ± standard error of the mean based on three independent experiments analyzed by unpaired two-tailed t-test and one-way ANOVA. ^*^P < 0.05, ^**^P < 0.01, ^***^P < 0.001, ^****^P < 0.0001, ns (not significant)

As we observed a reduced rate of glioma cell proliferation following DCX knockdown, we explored if apoptosis could be the determinant for this decelerated proliferation. qPCR and Western blot analyses of apoptotic proteins showed elevated levels of Bax and cleaved caspase-3 alongside a reduction in Bcl-2 expression and no significant change was observed in the total caspase-3 levels following DCX knockdown (Fig. 2A and B). An increase in caspase-3/7 activity was also detected (Fig. 2C).

Previous studies have validated the inhibitory effect of TMZ and gamma radiation on human glioma in vitro and in vivo (Whitehead et al. 2018). To further confirm whether DCX knockdown could enhance apoptosis, we subjected DCX knockdown cells to TMZ (50 µM) and γ-radiation (8 Gy) treatment for 24 h. As shown in (Fig. 2D), there was a noticeable increase in Bax and cleaved-caspase-3, accompanied by a decrease in Bcl-2 expression with no significant change in total caspase-3 levels. Notably, the irradiation treatment exhibited a more pronounced increase in Bax and cleaved-caspase-3 expression compared to the other groups. Next, flow cytometry analysis revealed a higher proportion of apoptotic (Annexin V and 7-AAD double positive) cells in the knockdown group treated with γ-radiation compared to the TMZ-treated and control group (Fig. 2E). In line with these findings, immunofluorescence staining showed that DCX knockdown in conjunction with TMZ and irradiation treatment increased cleaved caspase-3 expression (Fig. 2F). Collectively, these findings suggest that DCX knockdown may promote glioma cell apoptosis by enhancing the efficacy of TMZ and irradiation therapy.

### DCX depletion triggers mitochondria apoptosis in glioma cell

**Fig. 3:**
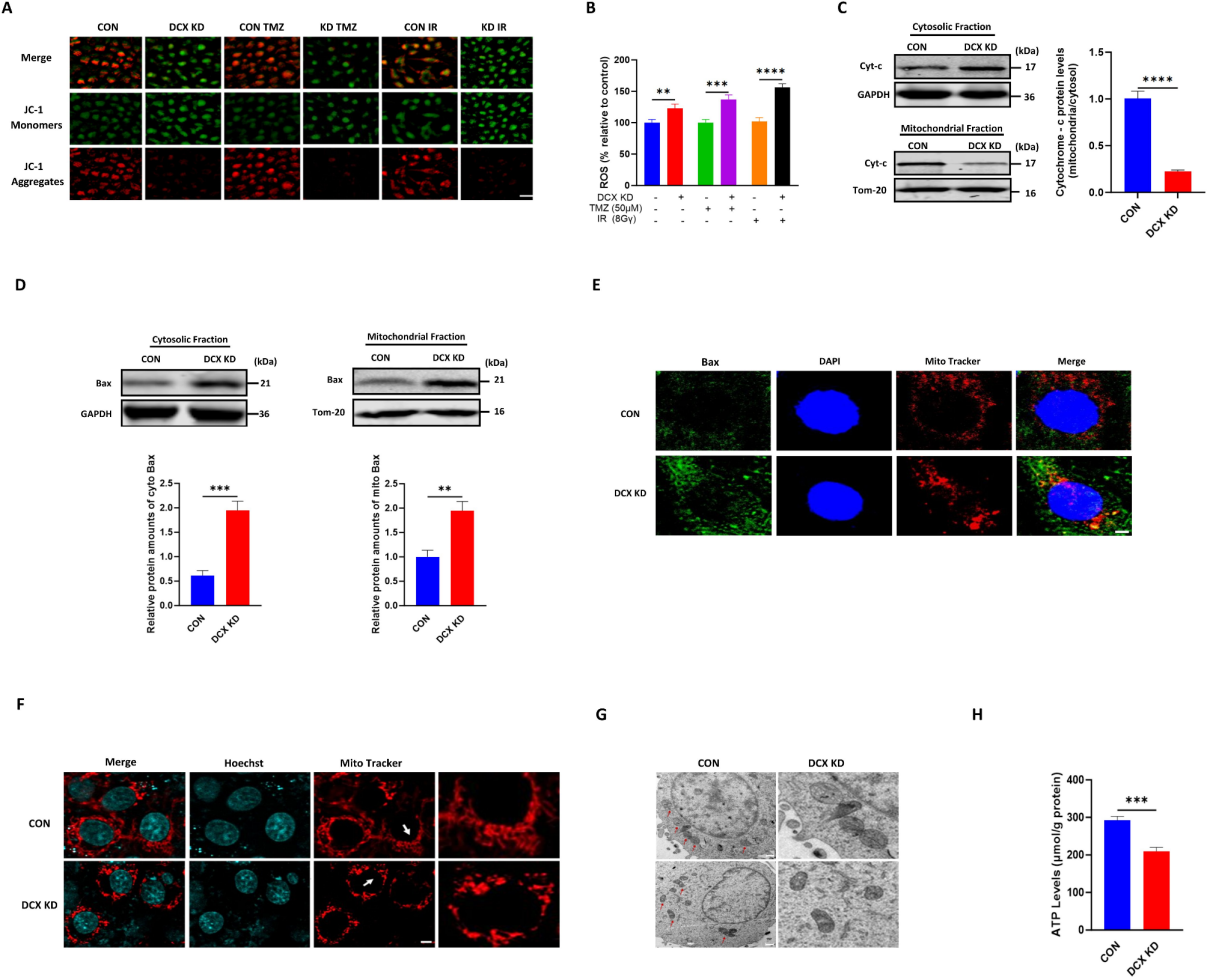
DCX depletion triggers mitochondria apoptosis in glioma cell. **(A)** Fluorescence microscopy images depicting the loss of MMP in DCX knockdown cells before and after treatment, as indicated by increased green fluorescence intensity of JC-1 monomers. Scale bar, 50 μm. **(B)** Measurement of intracellular ROS levels using fluorescent probe DCFH-DA, showing significant accumulation of ROS in DCX knockdown cells. **(C)** Release of cyt-C in the cytosol to mitochondria ratio measured by Western blotting. **(D)** Increased mitochondrial Bax expression levels in DCX knockdown cells compared to control analyzed by Western blotting. **(E)** Immunofluorescence analysis showing the translocation of Bax to mitochondria. **(F)** MitoTracker staining revealing morphological changes in mitochondria from filamentous to dot-shaped in DCX knockdown cells. **(G)** TEM images depicting elongated mitochondria in DCX knockdown cells and swollen mitochondria in the control group. **(H)** There was a significant reduction in cellular ATP levels in DCX knockdown cells compared to control. For all experiments, data represent the mean ± standard error of the mean based on three independent experiments analyzed by unpaired two-tailed t-test and one-way ANOVA. ^****^P < 0.0001, ^**^P < 0.01, ^***^P < 0.001

To elucidate the role of DCX knockdown in the intrinsic apoptotic pathway, we first assessed mitochondria membrane potential (MMP) using a JC-1 dye staining in U251 cells. The results indicated that MMP progressively decreased following DCX knockdown, with a particularly pronounced loss observed in the irradiation-treated group (Fig. 3A). Intracellular ROS levels were elevated after the DCX knockdown (Fig. 3B). The release of cytochrome c (Cyt-c) into the cytoplasm of DCX knockdown cells was confirmed by Western blotting (Fig. 3C).

Previous studies have demonstrated that Bax activation and translocation from cytosol to mitochondria in response to apoptotic stimuli are often suppressed in cancer cells (Liu et al. 2016). Western blot analysis of subcellular fractions revealed a significant increase in the Bax level within the mitochondrial fraction of the DCX knockdown group compared to the control group (Fig. 3D). Staining with a Bax antibody revealed a diffuse staining pattern in the DCX knockdown group, whereas the control group exhibited punctate staining (Fig. 3E). The shape and structural integrity of mitochondria are closely linked to their apoptotic function, with dot-shaped mitochondria morphology serving as a hallmark of apoptosis (Maes et al. 2019). MitoTracker staining showed that DCX knockdown led to noticeable changes in mitochondria morphology, with mitochondria transforming from filamentous to dot-shaped forms (Fig. 3F). Transmission electron microscopy (TEM) further revealed shrunken and elongated mitochondrial morphology in DCX knockdown cells compared to the control group (Fig. 3G). Additionally, ATP-based luminescence assays demonstrated a significant reduction in total cellular ATP levels following DCX knockdown (Fig. 3H). These findings suggest that DCX knockdown induces apoptosis in a mitochondria-dependent manner.

### DCX inhibits apoptosis through the Rho-A/Net-1/p38-MAPK pathway in glioma cell

**Fig. 4:**
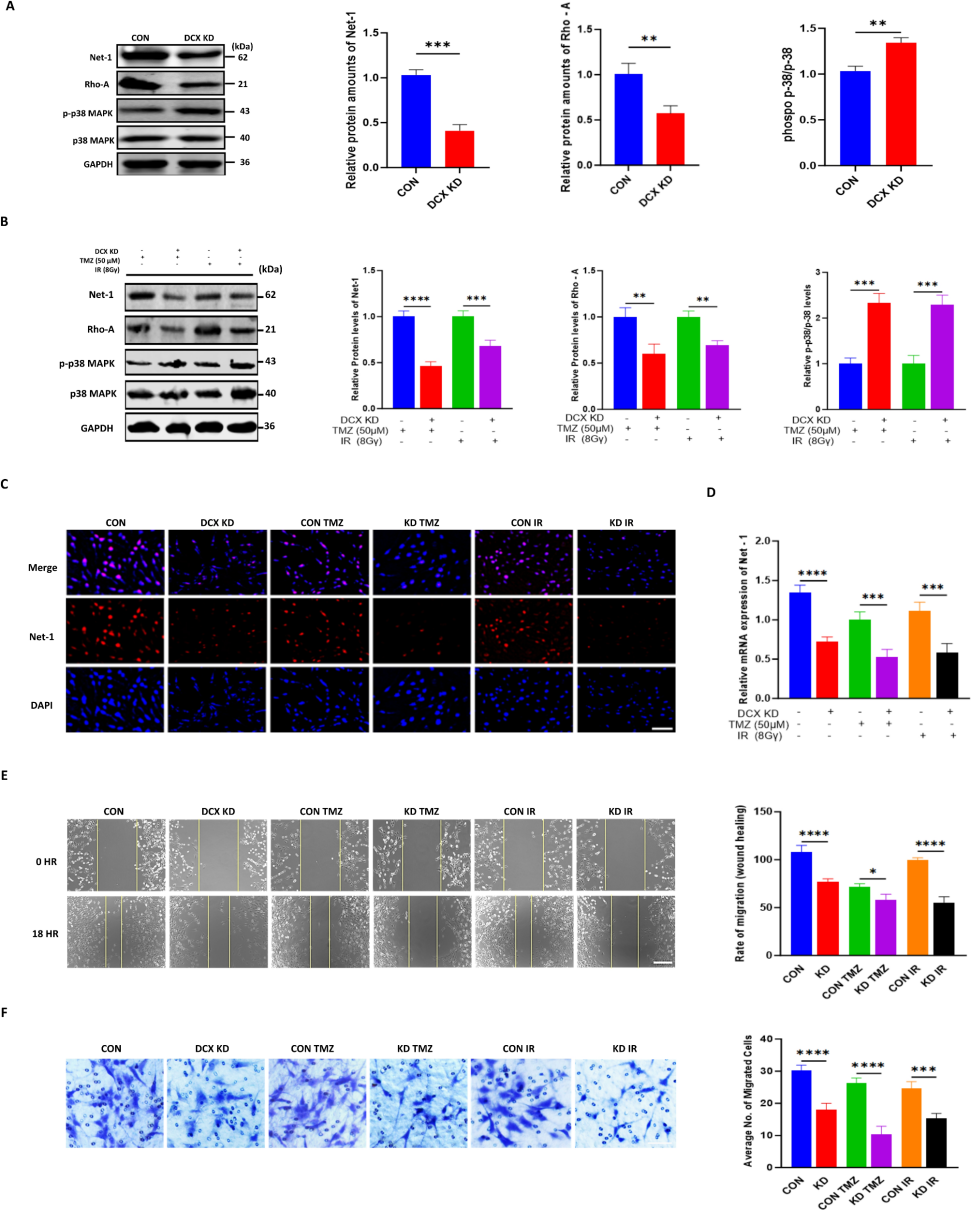
DCX inhibits apoptosis through the Rho-A/Net-1/p38-MAPK pathway in glioma cell. **(A)** Western blot analysis shows increased phosphorylated p38-MAPK levels and decreased Rho-A and Net-1 expression in the DCX knockdown group compared to the control. **(B)** Western blotting expression analysis of Net-1, Rho-A, p-38, and phosphorylated p-38 followed by DCX knockdown with chemotherapy and radiation in U251 cells (CON TMZ: Blue; KD TMZ: Red; CON IR: Green; KD IR: Purple). Analyses of protein were performed using Image J. **(C and D)** Detection of Net-1 levels by mRNA and immunostaining in U251 cells with knockdown and treatment. Scale bar, 20 μm. **(E)** Scratch wound healing shows the migration capacity reduction in the U251 cells with DCX knockdown and TMZ/IR treatment (CON: Blue; DCX KD: Red; CON TMZ: Green; KD TMZ: Purple; CON IR: Orange; KD IR: Black). **(F)** DCX knockdown cells with IR treatment had the lowest average number of migrating cells among the three groups in the transwell migration assay. For all experiments, data represent the mean ± standard error of the mean based on three independent experiments analyzed by unpaired two-tailed t-test and one-way ANOVA. ^*^P < 0.05, ^****^P < 0.0001, ^**^P < 0.01, ^***^P < 0.001

DCX is known for regulating cytoskeletal organization, however, its molecular processes related to apoptosis are unknown ^9^. Previous studies have highlighted the involvement of Rho-A, Net-1, and p38-MAPK in cancer proliferation, invasion, and cell death (Al-Koussa et al. 2020; Tu et al. 2010)^−^(Demuth et al. 2007; Yuan et al. 2013). To explore the underlying molecular mechanism, we probed for the expression of key cytoskeleton dynamics-related proteins Rho-A, Net-1, and p-38 MAPK signaling molecules. Western blot results showed DCX knockdown group exhibited significant downregulation of Net-1 and Rho-A expression and an upregulation of phosphorylated p38-MAPK while total p38-MAPK remained unchanged (Fig. 4A).

Furthermore, to check the inhibitory effects after silencing DCX, cells were treated individually with TMZ and γ-radiation for 24 h. As shown in (Fig. 4B), an obvious reduction in Rho-A, Net-1, and an increase in phosphorylated p38-MAPK levels were more distinctive in DCX knockdown groups treated with TMZ. This observation was supported by subsequent immunostaining and qPCR analyses, which demonstrated a significant decrease in Net-1 expression in the same treatment groups (Fig. 4C, D). Additionally, Transwell and wound healing assays were conducted to evaluate the effect of DCX depletion on glioma cell migration and invasion after exposing cells to TMZ and irradiation. The rate of migration and invasion was significantly suppressed in treated groups compared to respective controls (Fig. 4E, F). These findings suggest that DCX depletion might contribute both indirectly and directly to glioma cell apoptosis by disrupting Net-1 and Rho-A expression and activating the p38-MAPK signaling pathway.

### DCX silencing impedes tumor growth and increases apoptosis in vivo

**Fig. 5:**
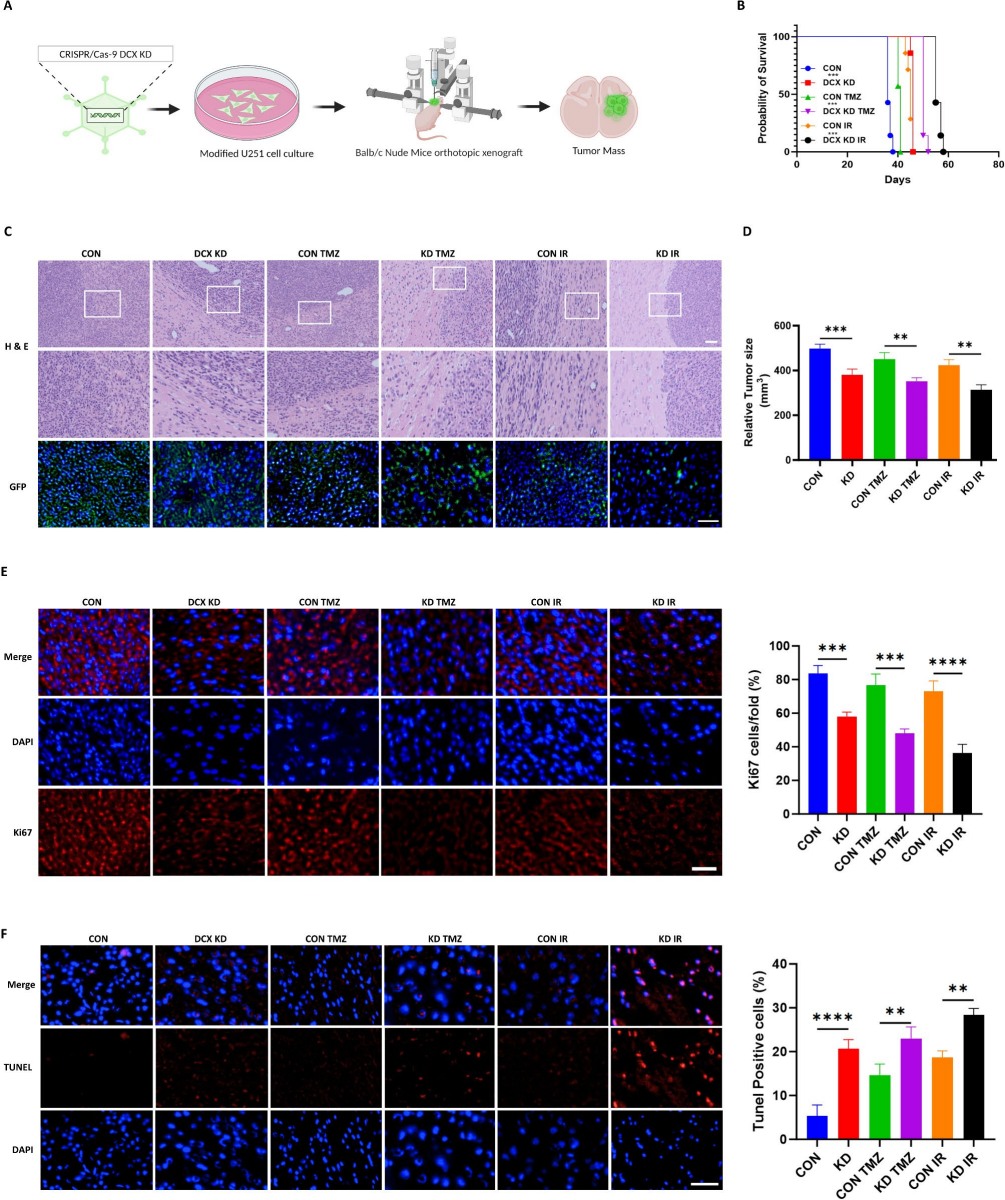
DCX silencing impedes tumor growth and increases apoptosis. *in vivo.* **(A)** Construction of a glioma orthotopic model using Balb/c nude mice with DCX-modified U251 cells. **(B)** Mouse survival is shown by Kaplan-Meier curves. The log-rank test was used to measure survival differences in the respective groups. **(C)** H&E staining and GFP tracking of tumor tissue in CON and DCX KD model mice. Scale bars = 20 μm. **(D)** Relative tumor size before and after treatment. **(E and F)** Ki67 immunostaining and TUNEL measurement of apoptosis of tumor specimens. Scale bars = 20 μm. ^****^P < 0.0001, ^***^P < 0.001, ^**^P < 0.01

**Fig. 6:**
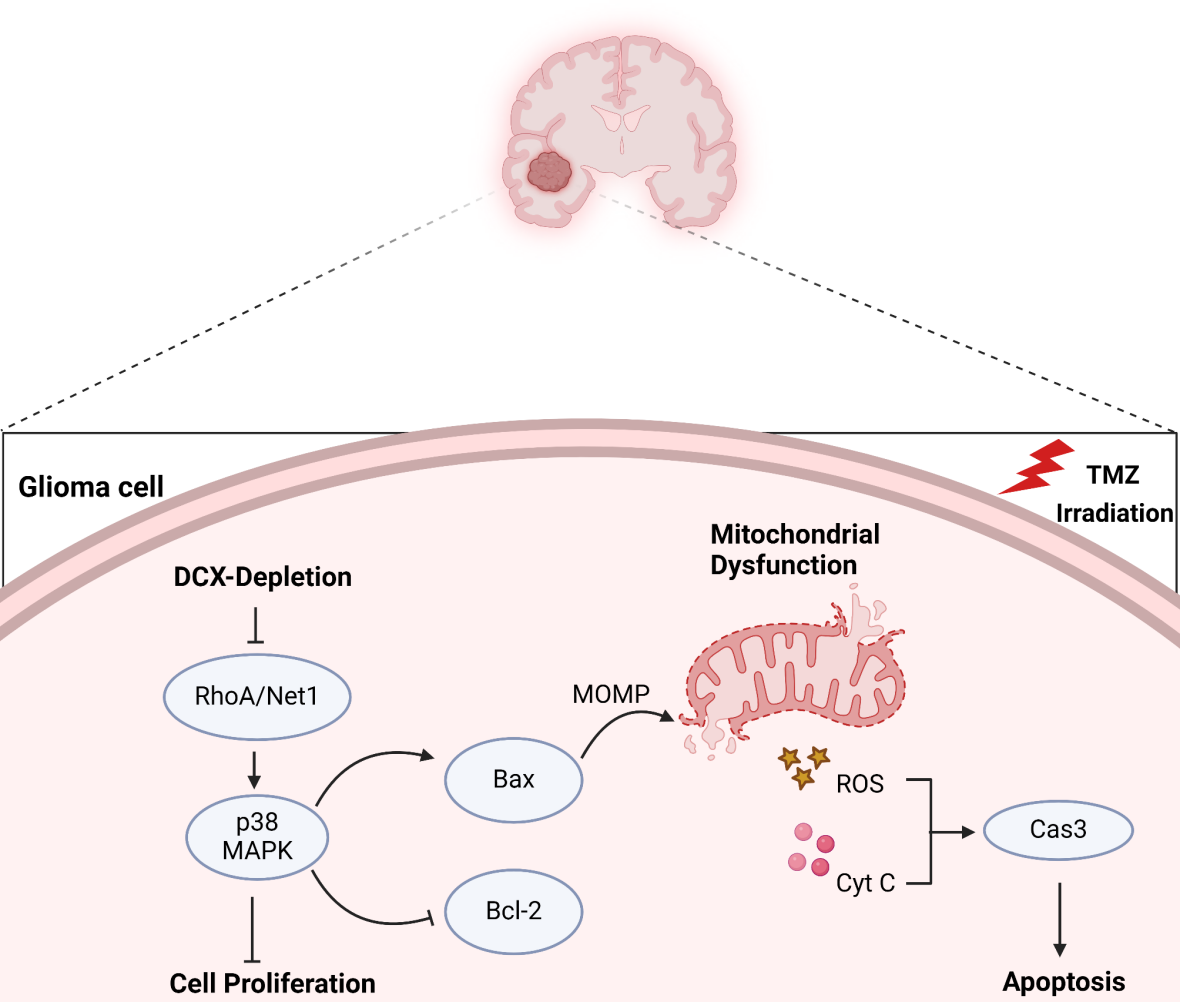
Schematic illustration of DCX knockdown-induced mitochondrial apoptosis mechanism in glioma. This illustration depicts the molecular mechanisms by which DCX knockdown triggers apoptosis in human glioma cells. The downregulation of Rho-A and Net-1 upon loss of DCX activates p38-MAPK signaling, resulting in mitochondrial dysfunction and apoptosis induction. Additionally, the figure shows the enhanced pro-apoptotic effects observed when DCX depletion is combined with TMZ and γ-radiation treatment

To assess the in vivo effects of DCX, we established an intracranial xenograft model using Balb/c nude mice by stereotaxically injecting them with CRISPR/Cas9 modified DCX knockdown U251 cells (Fig. 5A). Following a seven-day post-tumor implantation period, animals were randomly distributed into six groups (10 mice/group) for subsequent treatment and survival analysis. They were administrated with TMZ (50 mg/kg intraperitoneally) and γ-radiation (6 Gy at a rate of 2 Gy/min directed at a tumor site) for five days. Kaplan–Meier analysis showed that forty-five days post tumor cell implantation, DCX knockdown mice treated with TMZ, and irradiation individually survived significantly longer than the control groups (Fig. 5B). Histological and GFP immunofluorescence analysis revealed a marked reduction in the mice brain tumor in knockdown mice after TMZ and irradiation treatment compared to the controls (Fig. 5C, D). Ki67 immunostaining showed a notable impairment in the proliferation of glioma cells in vivo, particularly after irradiation, consistent with in vitro findings (Fig. 5E). Furthermore, a significant increase in TUNEL-positive cells was also observed in brain tumor slices (Fig. 5F). These findings demonstrated that DCX knockdown suppresses tumor growth and promotes apoptosis in vivo. (Fig. 6).

## Discussion

Human glioma is one of the most common invasive, proliferative, and aggressive tumors, which has a survival rate of < 5% with current therapeutic strategies, including surgery, radiotherapy, and chemotherapy (Davis 2018).

In line with these findings, we validated the role of DCX knockdown in glioma using CRISPR/Cas9 technology. Our results demonstrated that silencing DCX not only inhibited proliferation in U251 glioma cells (Fig. 1) but also reduced brain tumor growth in vivo. (Fig. 5). Exposure to chemotherapy and irradiation efficiently promotes DNA damage and causes the sensitization of glioma to cell death (Whitehead et al. 2018). Similarly, U251 cells with DCX knockdown when treated individually with conventional chemotherapy (TMZ) and radiotherapy (γ-radiation) showed an increase in apoptosis (Fig. 2).

In addition, we demonstrated that DCX depletion triggered Bax translocation to mitochondria and mitochondria dysfunction. The mitochondria-dependent pathway for apoptosis involves the release of cytochrome-c from mitochondria into the cytosol, either by suppressing anti-apoptotic members or activating pro-apoptotic members of the Bcl-2 family, leading to the activation of caspase-3 (Lopez and Tait 2015); Wolter et al. 1997). Interestingly, we found that DCX knockdown resulted in loss of mitochondria membrane potential, increase in intracellular ROS accumulation, and alteration in mitochondria morphology with a reduction in ATP, further contributing to apoptosis (Fig. 3). To the best of our knowledge, the present findings are the first experimental evidence to demonstrate that the suppression of DCX expression induces mitochondria-dependent apoptosis in glioma cells.

We also described a possible mechanistic function that DCX knockdown employs in glioma cells to trigger a cascade of events leading to apoptosis. Several studies suggest potential interactions and crosstalk between cytoskeleton dynamic regulatory proteins and apoptosis (Wattanathamsan and Pongrakhananon 2022). Net-1 is classically involved in cytoskeleton reorganization and activation of Rho-A (Becker et al. 2022; Fortin Ensign et al. 2013; Menon et al. 2013). Net-1 dysregulation has been implicated in tumor proliferation and resistance to apoptosis in various cancers, including glioma (Tu et al. 2010). Consistently, our results demonstrated that DCX knockdown downregulates the expression of Net-1/Rho-A proteins. Furthermore, chemotherapy, compared to irradiation, enhanced the suppressive effect of DCX downregulation on apoptosis. Given that MAPKs are central mediators of cell death and survival pathways (Grave et al. 2022), we further found that DCX depletion significantly activated the p38-MAPK pathway, especially in response to chemotherapy. When activated, p38-MAPK can modulate cell fate, potentially attenuating cellular proliferation (Yuan et al. 2013). These findings indicate that DCX knockdown in glioma could have a complex interplay between cytoskeleton dynamics and apoptotic signaling. Further research is needed to elucidate the precise mechanism.

While this study demonstrates the significant role of DCX knockdown in inhibiting glioma cell proliferation and promoting apoptosis, several limitations should be acknowledged. First, the findings are based on the U251 glioblastoma cell line, which may not fully represent the heterogeneity of glioma subtypes. Although we identified key apoptotic pathways affected by DCX knockdown, a more comprehensive understanding of the underlying mechanisms of cell death pathways is needed. Additionally, the use of an intracranial xenograft model in Balb/c nude mice, while informative, does not fully replicate the human tumor microenvironment and the complex interactions between glioma cells, which may influence chemotherapy and radiotherapy response. Future research should focus on employing a diverse glioma cell line panel and patient-derived xenografts model to evaluate the relevance of our findings across various glioma subtypes. Addressing the limitations identified in this study will be crucial for advancing the understanding of DCX’s role in glioma pathogenesis and its therapeutic significance.

In summary, our findings suggest that the CRISPR/Cas9-mediated disruption of DCX expression represents a potential strategy for inhibiting glioma proliferation.

Several studies have highlighted the involvement of microtubule-associated proteins (MAPs) in brain cancer biology, suggesting their potential as novel therapeutic targets for treating glioma (Breuzard et al. 2019; Verissimo et al. 2010; Wattanathamsan and Pongrakhananon 2022). DCX is a microtubule-associated protein at the crossroads between brain tumors and neurodevelopmental disorders, primarily known for its role in neuronal development and migration. Previous studies have demonstrated that DCX has an essential oncogenic function in glioma pathogenesis (Ayanlaja et al. 2020; Gleeson et al. 1999). Emerging evidence suggests that DCX plays a role in regulating cellular processes in glioma, especially increased expression of DCX, which was correlated with glioma cell invasiveness (Masui et al. 2008). Interfering with microtubule-associated protein expression can inhibit glioma cells growth and invasion (Verissimo et al. 2010), but the effect of DCX knockdown in glioma is totally unexplored. As DCX is essential for cell proliferation and migration, interrupting DCX would offer the potential to alter the infiltrative nature of glioma.

## Conclusions

Our study uncovers a novel role of Doublecortin (DCX) beyond its classical function in neuronal development, specifically in the regulation of mitochondria-dependent apoptosis in glioma. The knockdown of DCX enhances the therapeutic efficacy of chemotherapy and irradiation when administrated individually, both in vitro and in vivo. DCX depletion promotes apoptosis and reduces cell migration by downregulating Rho-A/Net-1 and activating p38-MPAK molecules. Targeting DCX presents a strategic approach to inhibit glioma growth and progression, highlighting its potential as a promising therapeutic target.

## Data Availability

No datasets were generated or analysed during the current study.

## Abbreviations

DCX: Doublecortin
TMZ: Temozolomide
IR: Irradiation
Cyto-C: Cytochrome-c
Net-1: Neuroepithelial Cell Transforming Gene 1
Rho-A: Ras homolog family member A
p38-MAPK: p38 Mitogen-Activated Protein Kinase
TUNEL: TdT-mediated dUTP-biotin nick end labeling
